# Activity strengths of cortical glutamatergic and GABAergic neurons are correlated with transgenerational inheritance of learning ability

**DOI:** 10.18632/oncotarget.19918

**Published:** 2017-08-04

**Authors:** Yulong Liu, Rongjing Ge, Xin Zhao, Rui Guo, Li Huang, Shidi Zhao, Sudong Guan, Wei Lu, Shan Cui, Shirlene Wang, Jin-Hui Wang

**Affiliations:** ^1^ Department of Pathophysiology, Bengbu Medical College, Anhui 233000, China; ^2^ Institute of Biophysics and University of Chinese Academy of Sciences, Beijing 100101, China; ^3^ Qingdao University, School of Pharmacy, Shandong 266021, China; ^4^ Department of Psychiatry, Northwestern University, Feinberg School of Medicine, Chicago, IL 60091, USA

**Keywords:** learning, memory, glutamate, GABA, neuron

## Abstract

The capabilities of learning and memory in parents are presumably transmitted to their offsprings, in which genetic codes and epigenetic regulations are thought as molecular bases. As neural plasticity occurs during memory formation as cellular mechanism, we aim to examine the correlation of activity strengths at cortical glutamatergic and GABAergic neurons to the transgenerational inheritance of learning ability. In a mouse model of associative learning, paired whisker and odor stimulations led to odorant-induced whisker motion, whose onset appeared fast (high learning efficiency, HLE) or slow (low learning efficiency, LLE). HLE male and female mice, HLE female and LLE male mice as well as HLE male and LLE female mice were cross-mated to have their first generation of offsprings, filials (F1). The onset of odorant-induced whisker motion appeared a sequence of high-to-low efficiency in three groups of F1 mice that were from HLE male and female mice, HLE female and LLE male mice as well as HLE male and LLE female mice. Activities related to glutamatergic neurons in barrel cortices appeared a sequence of high-to-low strength in these F1 mice from HLE male and female mice, HLE female and LLE male mice as well as HLE male and LLE female mice. Activities related to GABAergic neurons in barrel cortices appeared a sequence of low-to-high strength in these F1 mice from HLE male and female mice, HLE female and LLE male mice as well as HLE male and LLE female mice. Neuronal activity strength was linearly correlated to learning efficiency among three groups. Thus, the coordinated activities at glutamatergic and GABAergic neurons may constitute the cellular basis for the transgenerational inheritance of learning ability.

## INTRODUCTION

Associative learning is a common way for information acquisition. Associative memory is essential for logical reasoning and associative thinking [1, 2]. A traditional view is the transmission of cognitions from parents to their offspring, an idiom “like father like son”. There are two forms of transgenerational cognition. Parent cognition behaviors are directly transmitted to their offspring, such as specific odor-induced fear memory [3] and anti-predation behavior [4]. The parent's ability of learning and memory is transmitted to offspring [5–8]. The genetic codes and their epigenetic regulations are thought to be molecular bases for the transgenerational inheritance of learning ability and memory strength to specific events [9–17], in which genetical codes determine specific mRNAs and proteins to build up neural networks while epigenetic processes make chemical modifications of genetic codes to influence their expression levels. However, the cellular mechanism underlying this transgenerational inheritance of learning ability, such as associative learning, remains to be addressed. As neuronal plasticity occurs in associative memory [18–27], we study whether neuronal activity strengths in the brain areas of information storage are correlated with associative learning efficiency as one of cellular bases for the transgenerational inheritance of learning ability.

Interaction and balance between excitatory and inhibitory neurons are essential for brain codes to manage well-organized cognition [28]. As cortical glutamatergic and GABAergic neurons are recruited as associative memory cells for the storage of associated signals [22, 29–31], how these recruited glutamatergic and GABAergic neurons are refined for associative memory including transgenerational process remains unclear [17, 32]. Current reports show the upregulation of cortical glutamatergic neurons and the downregulation of GABAergic neurons during associative memory [17, 23]. Whether the coordinated plasticity among these neurons is correlated to the transgenerational inheritance of associative learning needs to be examined.

To these questions, we propose to study the transgenerational inheritance of associative learning ability by a mouse model of associative memory, odorant-induced whisker motion [17, 22, 30], and the correlation of coordinated activities at glutamatergic and GABAergic neurons with this transgenerational inheritance. The strategies to investigate these issues are given below. Paired whisker and odor stimulations led to odorant-induced whisker motion, whose fully expression appeared to be quick onset (high learning efficiency, HLE) and slow onset (low learning efficiency, LLE). HLE male and female mice, HLE female mice and LLE male mice, as well as HLE male mice and LLE female mice were cross-mated. In the first generation of offsprings, i.e., filials (F1) from three groups of parents, learning efficiency and barrel cortical neuronal activity were analyzed. The correlation between transgenerational learning ability and coordinated neuronal activities was quantified.

## RESULTS

### Odorant-induced whisker motion expresses differently in F1 mice from parents with different learning ability

Mice are trained by pairing whisker and odor stimulations, which leads to odorant-induced whisker motion [17, 22, 30]. When analyzing the strength of this odorant-induced whisker motion in day-by-day training, we can see that a fully establishment of this associative memory needs about either six training days in certain mice (high learning efficiency, HLE) or ten training days in others (low learning efficiency, LLE), as showed in Supplementary Figures 1–2. Mice used in present study were the first generation filials (F1) from the cross-mating of HLE male and female mice (HLE♂+HLE♀), HLE female and LLE male mice (HLE♀+LLE♂) as well as HLE male and LLE female mice (HLE♂+LLE♀). These three groups of F1 mice were trained by pairing whisker stimulus (WS) and odor stimulus (OS), including each training for twenty seconds, five times with two-hour intervals per day and consecutively ten days [22, 30].

**Figure 1:**
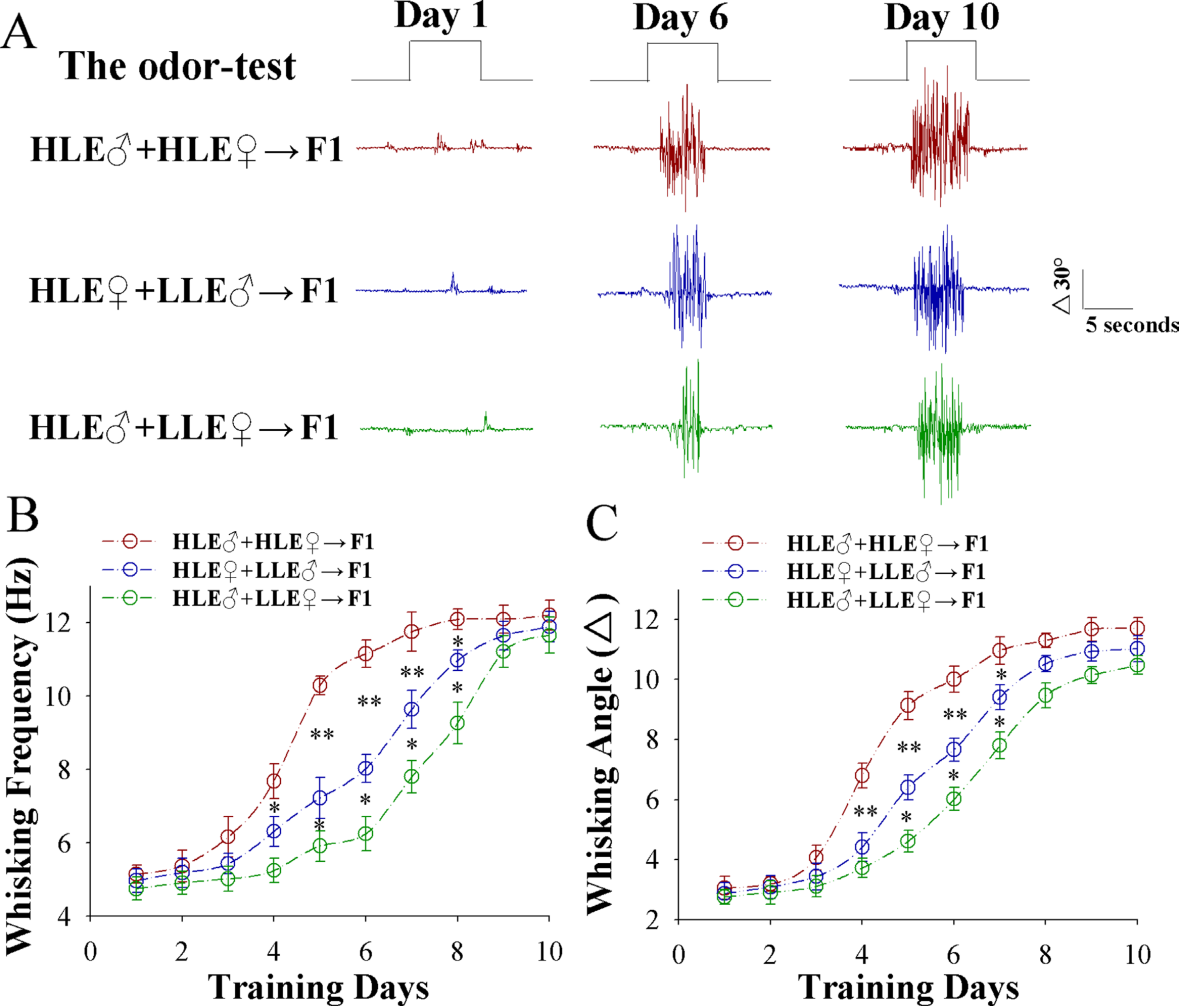
Paired whisker and odor stimulations lead to odorant-induced whisker motion more dominantly in the F1 mice cross-mated from the mice with high learning efficiency (HLE) (**A**) illustrates whisker motions in response to the odor-test (black traces on top) in CR-formation F1 mice from the cross-matings of HLE mice (HLE♂+HLE♀, red traces), HLE female mice and LLE male mice (HLE♀+LLE♂, blue) as well as HLE male mice and LLE female mice (HLE♂+LLE♀, green) at training days 0, 6 and 10, respectively. Calibration bars are 30^o^ and 5 seconds. (**B**) illustrates whisking frequencies in response to the odor-test versus training days in CR-formation F1 mice from the cross-matings of HLE mice (HLE♂+HLE♀, red symbols), HLE female mice and LLE male mice (HLE♀+LLE♂, blue) as well as HLE male mice and LLE female mice (HLE♂+LLE♀, green). (**C**) illustrates whisking angles in response to the odor-test versus training days in CR-formation F1 mice from the cross-matings of HLE mice (HLE♂+HLE♀, red traces), HLE female mice and LLE male mice (HLE♀+LLE♂, blue) as well as HLE male mice and LLE female mice (HLE♂+LLE♀, green). Asterisks indicate statistical comparisons between two neighboring groups. An asterisk shows *p* < 0.05, two asterisks show *p* < 0.01 and three asterisks show *p* < 0.001, in which the statistical comparisons are two-way ANOVA.

Odorant-induced whisker motion expresses after pairing the WS and OS. Its fully establishment appears variable at distinct training days among three groups of F1 mice (Figure 1A). Figure 1B shows whisking frequency in response to the OS versus training days in these F1 mice cross-mated from HLE♂+HLE♀ (red symbols, *n* = 8), HLE♀+LLE♂ (blue, *n* = 8) and HLE♂+LLE♀ (green, *n* = 8). Figure 1C shows whisking angles in response to the OS versus training days in these F1 mice from HLE♂+HLE♀ (red symbols), HLE♀+LLE♂ (blue) and HLE♂+LLE♀ (green *n* = 8 mice for each group). The efficiency of associative learning appears HLE in F1 mice that are filials of HLE male and female mice, middle learning efficiency (MLE) in F1 mice from HLE female and LLE male mice as well as LLE in F1 mice from HLE male and LLE female mice. This result indicates that parents with high learning ability are able to generate the offsprings with high learning ability, or vice versa, especially female in dominance.

To reveal the cellular mechanisms underlying the different efficiency of associative memory (i.e., odorant-induced whisker motion) in these F1 mice cross-mated from the parents with different learning ability, we analyzed the activity strengths of glutamatergic and GABAergic neurons in the barrel cortices from these F1 mice, where the associative memory cells were recruited [17, 22, 30].

### Barrel cortical glutamatergic neurons are upregulated in F1 mice with different learning efficiency

The transgenerational inheritance of associative learning, i.e., the high ability of associative learning in the F1 mice from the parents with high learning ability, or vice versa, is hypothetically based on the transgenerational inheritance in the activity strength of barrel cortical glutamatergic neurons. We examined this hypothesis at YFP-labeled glutamatergic neurons in layers II–III of barrel cortices from three groups of F1 mice with different learning abilities that were judged based on their whisker motions in response to the odor-test at training day six. As showed in Figure 1, HLE F1 mice are filials from HLE♂+HLE♀ mice, MLE F1 mice are filials from HLE♀+LLE♂ mice and LLE F1 mice are filials from HLE♂+LLE♀ mice. In a coronal direction of brain slices including barrel cortex, spontaneous excitatory postsynaptic currents (sEPSC) were recorded under the whole-cell voltage clamp to estimate excitatory synaptic transmission. The input-output curves of these neurons were measured under the current clamp to evaluate their ability to convert excitatory inputs into digital spikes. Spontaneous inhibitory postsynaptic currents (IPSC) were recorded to assess inhibitory synaptic function [22, 33].

**Figure 2:**
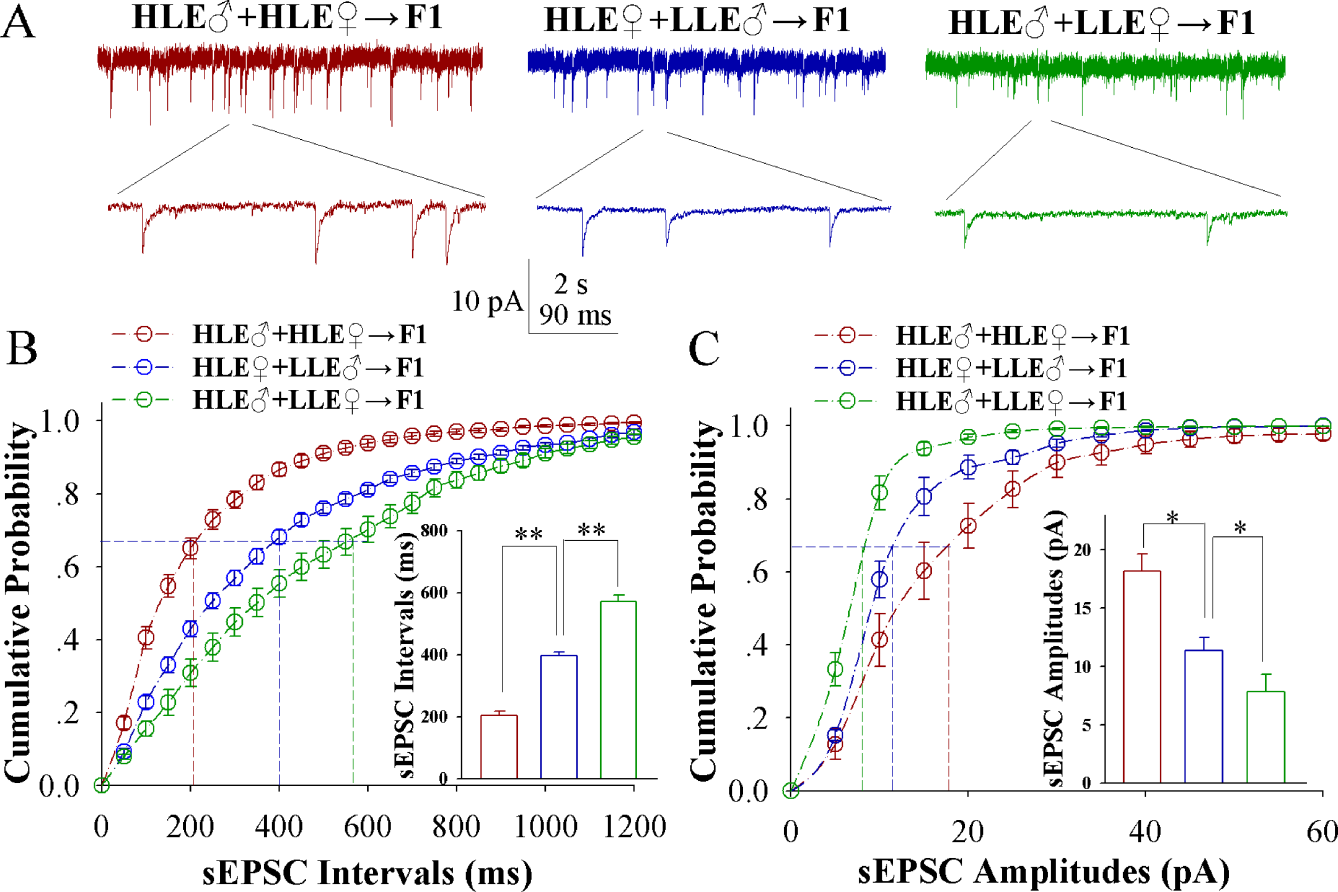
Excitatory synaptic transmission on barrel cortical glutamatergic neurons increases after pairing WS and OS, especially in the F1 mice with the high efficiency of odorant-induced whisker motion from the HLE parents Spontaneous excitatory postsynaptic currents (sEPSC) were recorded on the YFP-labeled glutamatergic neurons in cortical slices under voltage-clamp (holding potential at -70 mV) in presence of 10 μM bicuculline, in which three F1 groups were studied in training day 6. (**A**) illustrates sEPSCs recorded on the neurons in CR-formation F1 mice from cross-matings of HLE mice (HLE♂+HLE♀, red trace), HLE female mice and LLE male mice (HLE♀+LLE♂, blue) as well as HLE male mice and LLE female mice (HLE♂+LLE♀, green). Calibration bars are 10 pA, 2 second (top) and 90 ms (bottom). (**B**) shows cumulative probability versus sEPSC interval in the neurons from CR-formation F1 mice from the cross-matings of HLE mice (HLE♂+HLE♀, red symbols), HLE female mice and LLE male mice (HLE♀+LLE♂, blue) as well as HLE male mice and LLE female mice (HLE♂+LLE♀, green). Insert figure shows the comparisons of sEPSC intervals at 67% cumulative probability from three groups of mice (*n* = 15 neurons from 8 mice for each group). (**C**) shows cumulative probability versus sEPSC amplitudes in the neurons from CR-formation F1 mice from the cross-matings of HLE mice (HLE♂+HLE♀, red symbols), HLE female mice and LLE male mice (HLE♀+LLE♂, blue) as well as HLE male mice and LLE female mice (HLE♂+LLE♀, green). Insert figure denotes the comparisons of sEPSC amplitudes at 67% cumulative probability from three groups of mice (*n* = 15 neurons from 8 mice for each group). An asterisk shows *p* < 0.05, two asterisks show *p* < 0.01 and three asterisks show *p* < 0.001, in which the statistical comparisons are one-way ANOVA.

In comparison of sEPSCs from these F1 mice (Figure 2A), the increases of excitatory synaptic transmission on barrel cortical glutamatergic neurons appear a high-to-low sequence in HLE F1 mice (red trace), MLE F1 mice (blue) and LLE F1 mice (green). Figure 2B shows cumulative probability versus sEPSC intervals on glutamatergic neurons from HLE F1 mice (red symbols), MLE F1 mice (blues) and LLE F1 mice (greens; *n* = 15 cells from 8 mice in each group). sEPSC intervals at 67% cumulative probability are 203.13 ± 15.35 ms on glutamatergic neurons from HLE F1 mice (red bar in insert figure), 396.45 ± 12.38 ms from MLE F1 mice (blue) and 571.62 ± 20.43 ms from LLE F1 mice (green; asterisk, *p* < 0.05; two asterisks, *p* < 0.01 and three asterisks, *p* < 0.001). Figure 2C shows cumulative probability versus sEPSC amplitudes on glutamatergic neurons from HLE F1 mice (red symbols), MLE F1 mice (blues) and LLE F1 mice (greens; *n* = 15 cells from 8 mice for each group). sEPSC amplitudes at 67% cumulative probability are 18.15 ± 1.52 pA on glutamatergic neurons from HLE F1 mice (red bar in insert figure), 11.35 ± 1.14 pA from MLE F1 mice (blue) and 7.83 ± 1.48 pA from LLE F1 mice (green; asterisk, *p* < 0.05; two asterisks, *p* < 0.01 and three asterisks, *p* < 0.001). Thus, the neural substrates for associative memory (odorant-induced whisker motion) may be based on the functional upregulation of excitatory synaptic transmission on the barrel cortical glutamatergic neurons and the strength of the upregulated synaptic transmission is associated to learning efficiency in F1 mice.

**Figure 3:**
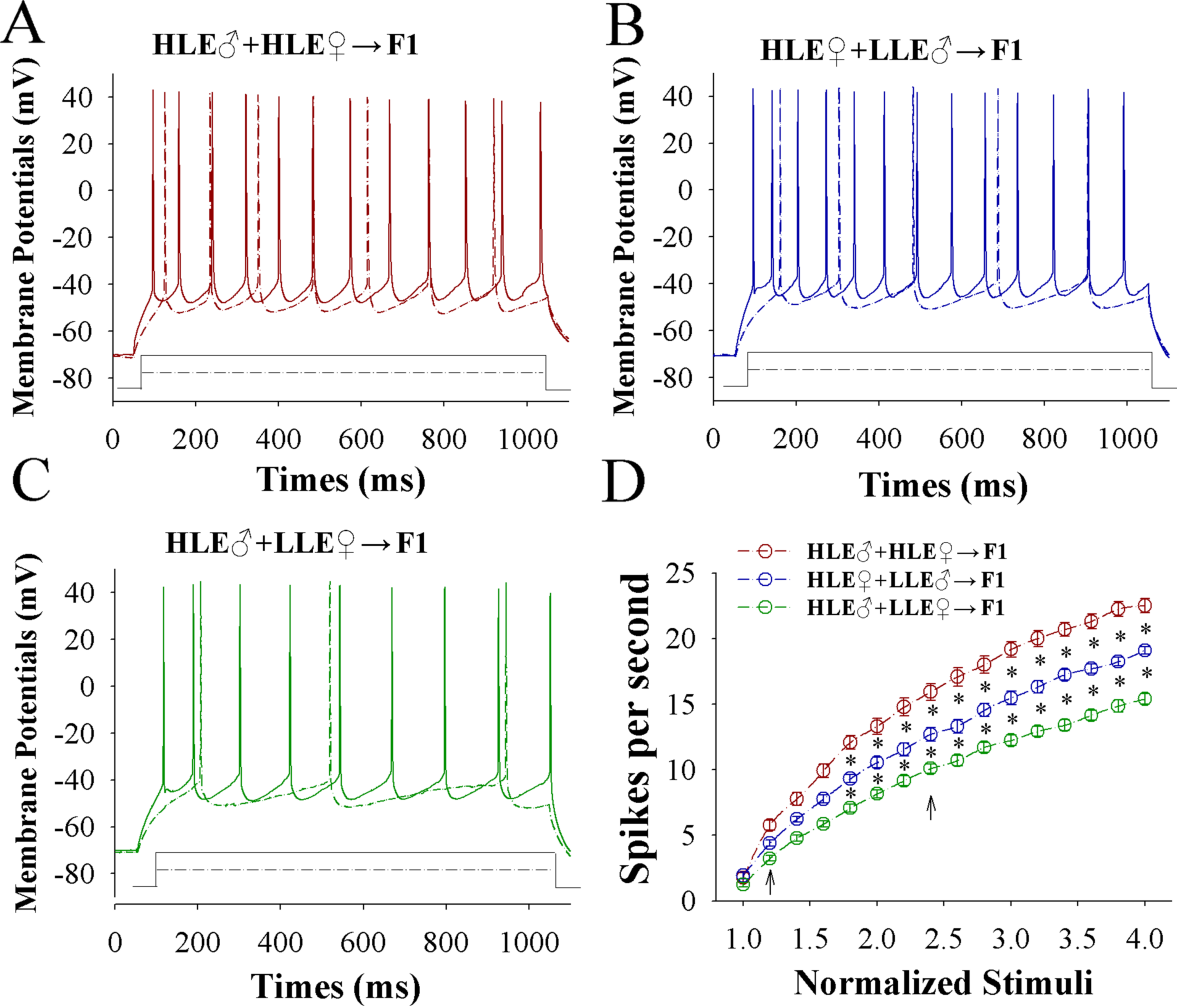
The capability to encode spikes on barrel cortical glutamatergic neurons increases after pairing WS and OS, especially in F1 mice with the high efficiency of odorant-induced whisker motion from the HLE parents Sequential spikes were induced by depolarization pulses under current-clamp recording on YFP-labeled glutamatergic neurons in cortical slices. (**A**) illustrates the spikes induced by two-steps of depolarization pulse on the neurons in a CR-formation F1 mouse from the cross-mating of HLE mice (HLE♂+HLE♀). (**B**) illustrates the spikes induced by two-steps of depolarization pulse on the neurons in a CR-formation F1 mouse from the cross-mating of HLE female mice and LLE male mice (HLE♀+LLE♂). (**C**) shows the spikes induced by two-steps of depolarization pulse on the neurons in a CR-formation F1 mouse from cross-matings of HLE male mice and LLE female mice (HLE♂+LLE♀). Solid lines and dash lines present the spikes induced correspondent depolarization pulses, respectively. (**D**) shows spikes per second versus normalized stimuli in F1 mice from cross-matings of HLE mice (HLE♂+HLE♀, red symbols), HLE female mice and LLE male mice (HLE♀+LLE♂, blue) as well as HLE male mice and LLE female mice (HLE♂+LLE♀, green; *n* = 15 neurons from 8 mice for each group). Asterisks indicate statistical comparisons between two neighboring groups. An asterisk shows *p* < 0.05, two asterisks show *p* < 0.01 and three asterisks show *p* < 0.001, in which the statistical comparisons are two-way ANOVA.

The capability of glutamatergic neurons to convert excitatory input into spikes appears upregulated in CR-formation mice (Figure 3A–3C) with a high-to-low sequence from HLE F1, MLE F1 and LLE F1 mice. Figure 3D shows spikes per second versus normalized stimuli in barrel cortical glutamatergic neurons from HLE F1 mice (red symbols), MLE F1 mice (blue) and LLE F1 mice (green), in which spikes per second are statistically different (*n* = 15 cells from 8 mice for each group; asterisk, *p* < 0.05 and two asterisks, *p* < 0.01). Associative learning upregulates the capability of barrel cortical glutamatergic neurons to convert excitatory inputs into digital spikes for the information storage, especially in HLE F1 mice cross-mated from HLE parent mice.

**Figure 4:**
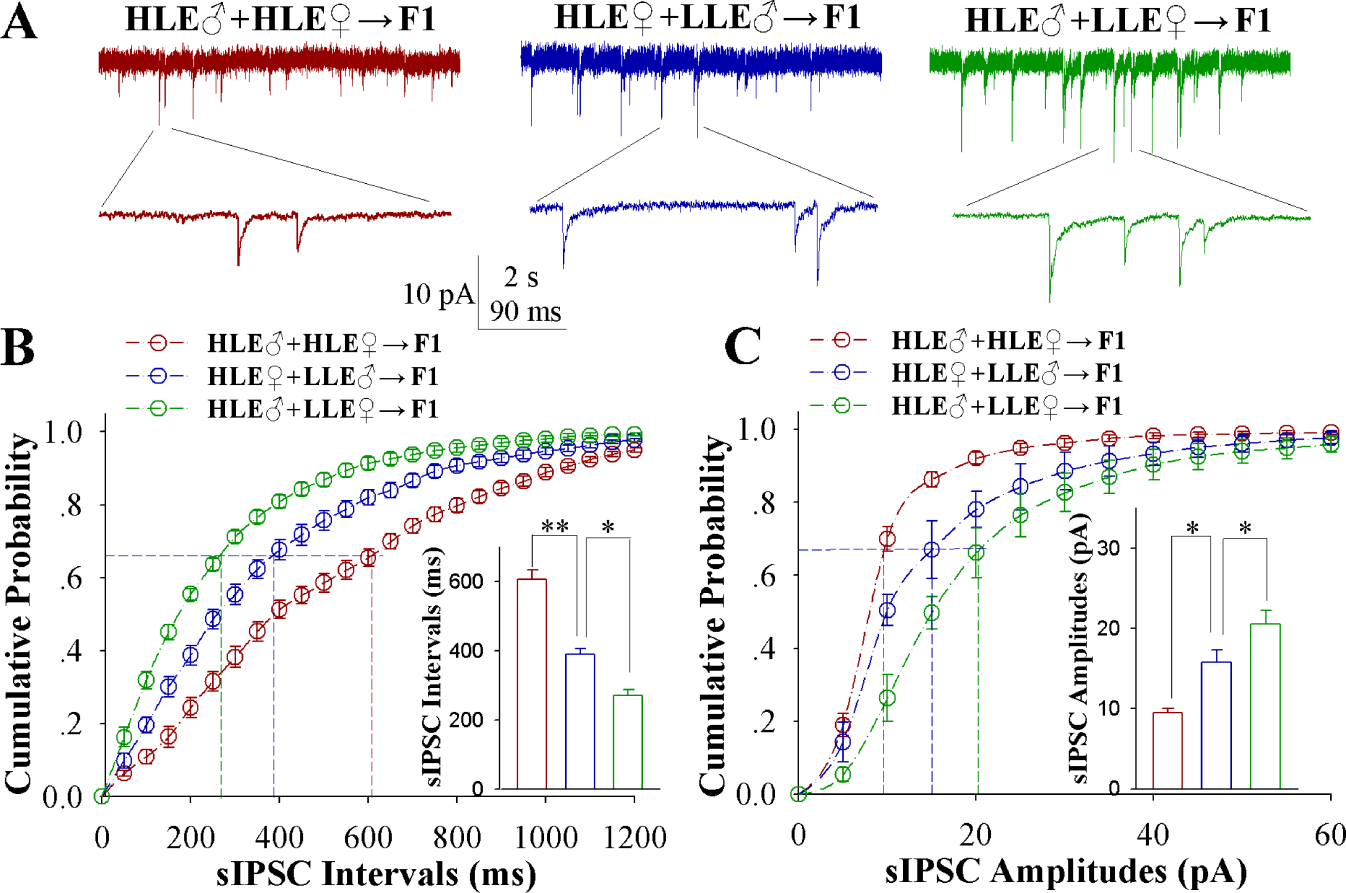
Inhibitory synaptic transmission on barrel cortical glutamatergic neurons decreases after pairing WS and OS, especially in the F1 mice with the high efficiency of odorant-induced whisker motion from the HLE parents Spontaneous inhibitory postsynaptic currents (sIPSC) were recorded on YFP-labeled glutamatergic neurons in cortical slices under voltage-clamp (holding potential at -65 mV) in presence of 10 μM CNQX and 40 μM D-AP5. (**A)** shows sIPSCs recorded on the neurons in CR-formation F1 mice from cross-matings of HLE mice (HLE♂+HLE♀, red trace), HLE female mice and LLE male mice (HLE♀+LLE♂, blue) as well as HLE male mice and LLE female mice (HLE♂+LLE♀, green). Bottom traces are the expanded waveforms selected from top traces. Calibration bars are 10 pA, 2 second (top) and 90 ms (bottom). (**B)** illustrates cumulative probability versus sIPSC intervals on the neurons in CR-formation F1 mice from cross-matings of HLE mice (HLE♂+HLE♀, red symbols), HLE female mice and LLE male mice (HLE♀+LLE♂, blue) as well as HLE male mice and LLE female mice (HLE♂+LLE♀, green). Inserted figure shows the comparisons of sIPSC intervals at 67% cumulative probability from three groups of mice (*n* = 15 neurons from nine mice for each group). (**C)** shows cumulative probability versus sIPSC amplitudes on the neurons in CR-formation F1 mice from cross-matings of HLE mice (HLE♂+HLE♀, red symbols), HLE female mice and LLE male mice (HLE♀+LLE♂, blue) as well as HLE male mice and LLE female mice (HLE♂+LLE♀, green). Inserted figure denotes the comparisons of sIPSC amplitudes at 67% cumulative probability from three groups of mice (*n* = 15 neurons from 8 mice for each group). An asterisk shows *p* < 0.05, two asterisks show *p* < 0.01 and three asterisks show *p* < 0.001, in which the statistical comparisons are one-way ANOVA.

The effect of associative learning on inhibitory synaptic function in barrel cortical glutamatergic neurons is showed in Figure 4. sIPSCs on barrel cortical glutamatergic neurons in CR-formation mice appears downregulated in a high-to-low sequence from HLE F1, MLE F1 and LLE F1 mice (Figure 4A). Figure 4B illustrates cumulative probability versus sIPSC intervals on glutamatergic neurons from HLE F1 mice (red symbols), MLE F1 mice (blues) and LLE F1 mice (greens; *n* = 15 cells from 8 mice in each group). sIPSC intervals at 67% cumulative probability are 607.25 ± 25.50 ms on glutamatergic neurons from HLE F1 mice (red bar in insert figure), 390.27 ± 16.65 ms from MLE F1 mice (blue) and 271.54 ± 16.81 ms from LLE F1 mice (green; asterisk, *p* < 0.05; two asterisks, *p* < 0.01 and three asterisks, *p* < 0.001). Figure 4C shows cumulative probability versus sIPSC amplitude on glutamatergic neurons from HLE F1 mice (red symbols), MLE F1 mice (blues) and LLE F1 mice (greens; *n* = 15 cells from 8 mice for each group). sIPSC amplitudes at 67% cumulative probability are 9.47 ± 0.55 pA on glutamatergic neurons from HLE F1 mice (red bar in insert figure), 15.83 ± 1.51 pA from MLE F1 mice (blue) and 20.58 ± 1.72 pA from LLE F1 mice (green; asterisk, *p* < 0.05; two asterisks, *p* < 0.01 and three asterisks, *p* < 0.001). Therefore, the neuronal substrates for associative memory may also be based on the functional downregulation of inhibitory synaptic transmission on the barrel cortical glutamatergic neurons, and the strength of the downregulated synaptic transmission is associated with learning efficiency in F1 mice.

In summary, associative learning by pairing whisker and odor signals can lead to the upregulations of the excitatory synaptic transmission and the encoding capability as well as the downregulation of GABAergic synaptic transmission on glutamatergic neurons in the barrel cortex, especially in the F1 mice with high learning efficiency. These changes may facilitate the recruitment and refinement of barrel cortical glutamatergic neurons as associative memory cells. We subsequently investigated activity strength at barrel cortical inhibitory neurons after associative learning.

### Barrel cortical GABAergic neurons are downregulated in F1 mice with different learning efficiency

In terms of plasticity at barrel cortical GABAergic neurons during associative learning, we have analyzed their excitatory synaptic inputs and ability to convert excitatory inputs into digital spikes at GFP-labeled GABAergic neurons in HLE, MLE and LLE F1 mice. sEPSCs were recorded to assess their reception of the excitatory synaptic transmission. The input-output curves of these neurons were measured to evaluate the ability to convert excitatory inputs into digital spikes [22, 33].

**Figure 5:**
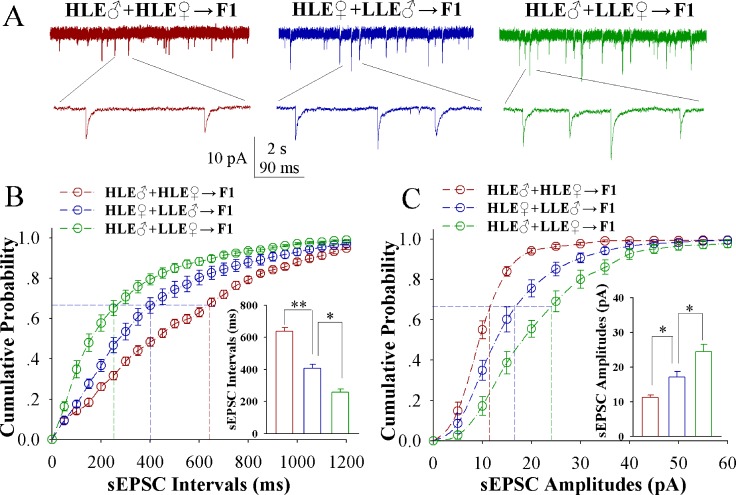
Excitatory synaptic transmission on barrel cortical GABAergic neurons decreases after pairing WS and OS, especially in F1 mice with the high efficiency of odorant-induced whisker motion from HLE parents Spontaneous excitatory postsynaptic currents (sEPSC) were recorded on the GFP-labeled GABAergic neurons in cortical slices under voltage-clamp (holding potential at -65 mV) in presence of 10 μM bicuculline. (**A**) shows sEPSCs recorded on the neurons in CR-formation F1 mice from cross-matings of HLE mice (HLE♂+HLE♀, red traces), HLE female mice and LLE male mice (HLE♀+LLE♂, blue) as well as HLE male mice and LLE female mice (HLE♂+LLE♀, green).. Bottom traces are the expanded waveforms selected from top traces. Calibration bars are 10 pA, 2 second (top) and 90 ms (bottom). (**B**) shows cumulative probability versus sEPSC intervals in the neurons from CR-formation F1 mice from cross-matings of HLE mice (HLE♂+HLE♀, red symbols), HLE female mice and LLE male mice (HLE♀+LLE♂, blue) as well as HLE male mice and LLE female mice (HLE♂+LLE♀, green). Inserted figure shows the comparisons of sEPSC intervals at 67% cumulative probability from three groups of mice (*n* = 15 neurons from 8 mice for each group). (**C**) shows cumulative probability versus sEPSC amplitudes in the neurons from CR-formation F1 mice from cross-matings of HLE mice (HLE♂+HLE♀, red symbols), HLE female mice and LLE male mice (HLE♀+LLE♂, blue) as well as HLE male mice and LLE female mice (HLE♂+LLE♀, green). Insert figure denotes the comparisons of sIPSC amplitudes at 67% cumulative probability from three groups of mice (*n* = 15 neurons from 8 mice for each group). An asterisk shows *p* < 0.05, two asterisks show *p* < 0.01 and three asterisks show *p* < 0.001, in which the statistical comparisons are one-way ANOVA.

In comparisons of sEPSCs from F1 mice (Figure 5A), excitatory synaptic transmission on barrel cortical GABAergic neurons appears decreased in a high-to-low sequence from HLE F1 mice (red trace), MLE F1 mice (blue) and LLE F1 mice (green). Figure 5B illustrates cumulative probability versus sEPSC intervals on GABAergic neurons from HLE F1 mice (red symbols), MLE F1 mice (blues) and LLE F1 mice (greens; *n* = 15 cells from 8 mice in each group). sEPSC intervals at 67% cumulative probability are 637.36 ± 23.22 ms on GABAergic neurons from HLE F1 mice (red bar in insert figure), 406.18 ± 25.21 ms from MLE F1 mice (blue) and 258.60 ± 17.56 ms from LLE F1 mice (green; asterisk, *p* < 0.05; two asterisks, *p* < 0.01; three asterisks, *p* < 0.001). Figure 5C illustrates cumulative probability versus sEPSC amplitudes on GABAergic neurons from HLE F1 mice (red symbols), MLE F1 mice (blue) and LLE F1 mice (green; *n* = 15 cells from 8 mice for each group). sEPSC amplitudes at 67% cumulative probability are 11.24 ± 0.71 pA on GABAergic neurons from HLE F1 mice (red bar in insert figure), 17.11 ± 1.62 pA from MLE F1 mice (blue) and 24.47 ± 2.12 pA from LLE F1 mice (green; asterisk, *p* < 0.05; two asterisks, *p* < 0.01 and three asterisks, *p* < 0.001). Thus, the neural substrates for odorant-induced whisker motion may be based on the functional downregulation of excitatory synaptic transmission on barrel cortical GABAergic neurons, and the strength of the downregulated synaptic function is associated with learning efficiency.

**Figure 6:**
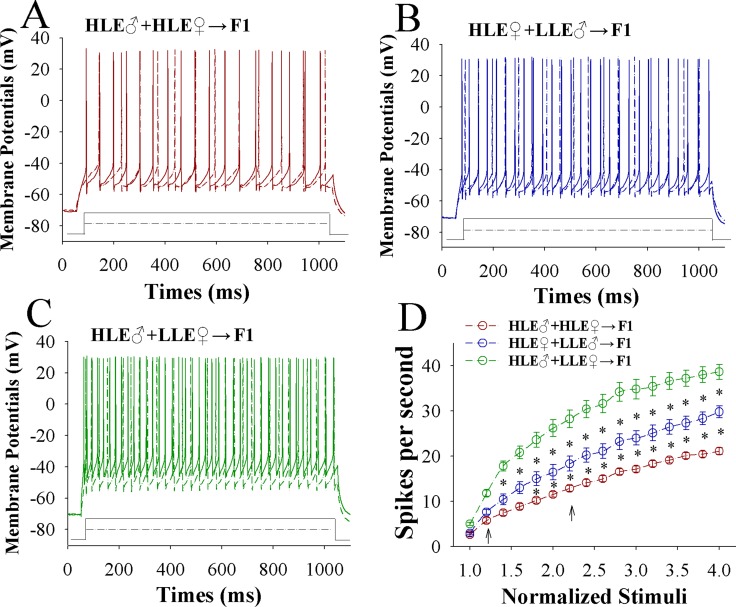
The capability to encode spikes on barrel cortical GABAergic neurons decreases after pairing WS and OS, especially in F1 mice with the high efficiency of odorant-induced whisker motion from the HLE parents Sequential spikes were induced by depolarization pulse under current-clamp recording on GFP-labeled GABAergic neurons in cortical slices. (**A**) shows the spikes induced by two-steps of depolarization pulse on the neurons in a CR-formation F1 mouse from the cross-matings of HLE mice (HLE♂+HLE♀). (**B**) shows the spikes induced by two-steps of depolarization pulse on the neurons in a CR-formation F1 mouse from the cross-matings of HLE female mice and LLE male mice (HLE♀+LLE♂). (**C**) shows the spikes induced by two-steps of depolarization pulse on the neurons in a CR-formation F1 mouse from cross-matings of HLE male mice and LLE female mice (HLE♂+LLE♀). Solid lines and dash lines present the spikes induced correspondent depolarization pulses, respectively. (**D**) shows spikes per second versus normalized stimuli in F1 mice from cross-matings of HLE mice (HLE♂+HLE♀, red symbols), HLE female mice and LLE male mice (HLE♀+LLE♂, blue) as well as HLE male mice and LLE female mice (HLE♂+LLE♀, green; *n* = 15 neurons from 8 mice for each group). Asterisks indicate statistical comparisons between two neighboring groups. An asterisk shows *p* < 0.05, two asterisks show *p* < 0.01 and three asterisks show *p* < 0.001, in which the statistical comparisons are two-way ANOVA.

The ability of GABAergic neurons to convert excitatory input into spikes appears downregulated in a high-to-low sequence from HLE F1 mice (red traces), MLE F1 mice (blue) and LLE F1 mice (green in Figure 6A–6C). Figure 6D shows spikes per second versus normalized stimuli in barrel cortical GABAergic neurons from HLE F1 mice (red symbols), MLE F1 mice (blues) and LLE F1 mice (greens), in which spikes per second are statistically different (*n* = 10 cells from 8 mice for each group; asterisk, *p* < 0.05 and two asterisks, *p* < 0.01). Associative learning downregulates the ability of barrel cortical GABAergic neurons to convert excitatory inputs into digital spikes for information storage, especially in F1 mice with high learning efficiency. The downregulations of the encoding ability in GABAergic neurons and their output synapse activities may facilitate the recruitment and refinement of glutamatergic neurons in the barrel cortex after associative learning.

**Figure 7:**
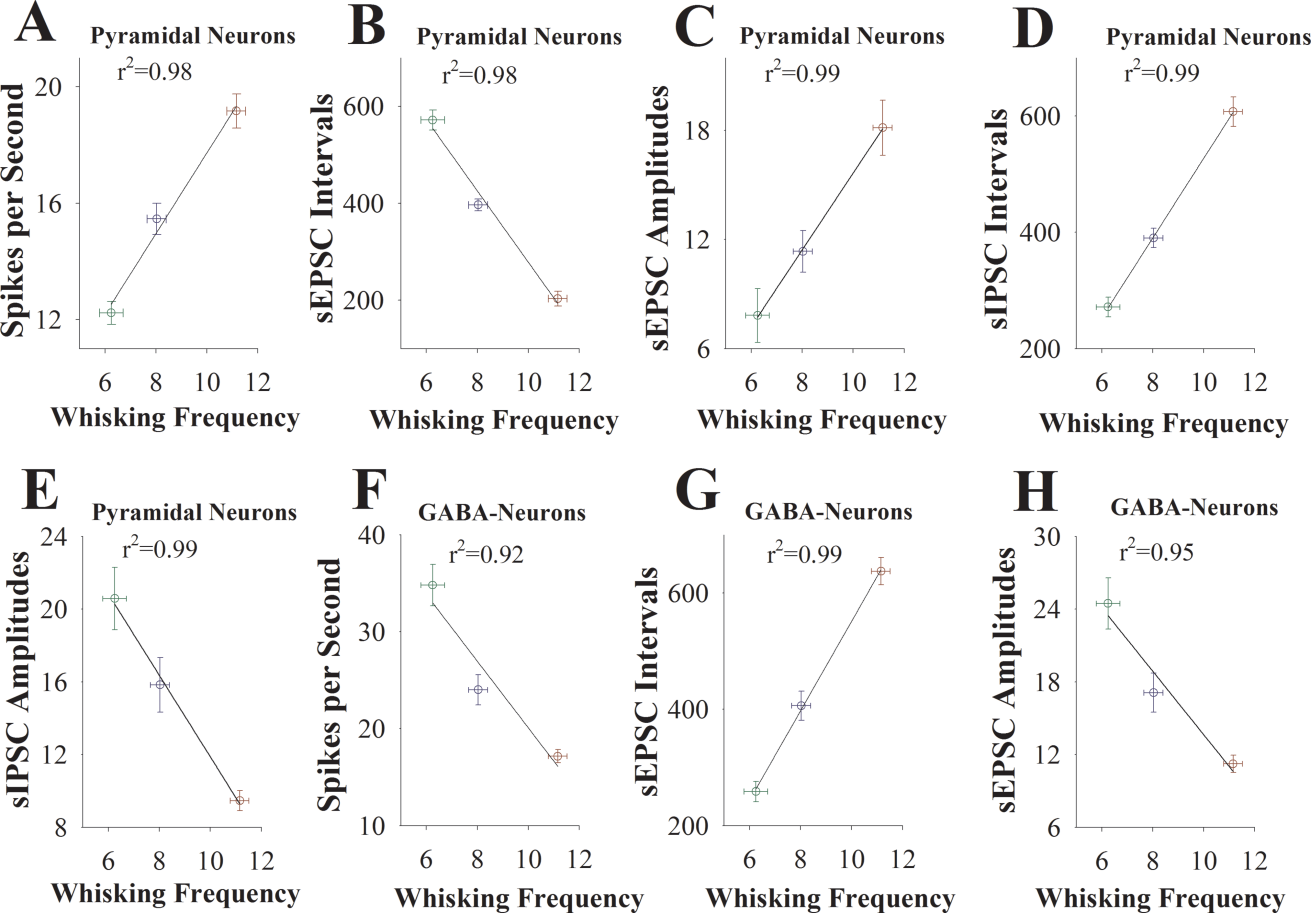
The activity strengths of barrel cortical glutamatergic and GABAergic neurons are linearly correlated with the efficiency of odorant-induced whisker motion in F1 mice (**A**) shows a correlation between spike per second on glutamatergic neurons and whisking frequency induced by the odor-test. (**B**) illustrates a correlation between sEPSC intervals on glutamatergic neurons and whisking frequency. (**C**) shows a correlation between sEPSC amplitudes on glutamatergic neurons and whisking frequency. (**D**) shows a correlation between sIPSC intervals on glutamatergic neurons and whisking frequency. (**E**) illustrates a correlation between sIPSC amplitudes on glutamatergic neurons and whisking frequency. (**F**) illustrates a correlation between spike per second on GABAergic neurons and whisking frequency. (**G**) illustrates a correlation between sEPSC intervals on GABAergic neurons and whisking frequency. (**H**) illustrates a correlation between sEPSC amplitudes on GABAergic neurons and whisking frequency. Data points for F1 mice that were from HLE male and female parents are red symbols. Data points for F1 mice that were from HLE female and LLE male are blue symbols. Data points for F1 mice that were from HLE male and LLE female are green symbols.

**Figure 8:**
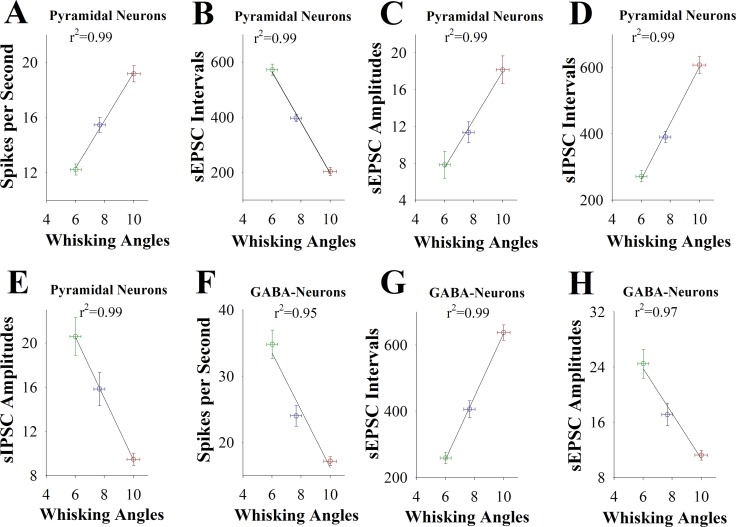
The activity strengths of barrel cortical glutamatergic and GABAergic neurons are linearly correlated with the efficiency of odorant-induced whisker motion in F1 mice (**A**) shows a correlation between spike per second on glutamatergic neurons and whisking angles induced by the odor-test. (**B**) illustrates a correlation between sEPSC intervals on glutamatergic neurons and whisking angles. (**C**) shows a correlation between sEPSC amplitude on glutamatergic neurons and whisking angles. (**D**) illustrates a correlation between sIPSC intervals on glutamatergic neurons and whisking angles. (**E**) shows a correlation between sIPSC amplitudes on glutamatergic neurons and whisking angles. (**F**) illustrates a correlation between spike per second on GABAergic neurons and whisking angles. (**G**) illustrates a correlation between sEPSC intervals on GABAergic neurons and whisking angles. (**H**) illustrates a correlation between sEPSC amplitudes on GABAergic neurons and whisking angles. Data points for F1 mice that were from HLE male and female parents are red symbols. Data points for F1 mice that were from HLE female and LLE male are blue symbols. Data points for F1 mice that were from HLE male and LLE female are green symbols.

Moreover, if the upregulation of glutamatergic neurons and the downregulation of GABAergic neurons in the barrel cortex is associated with odorant-induced whisker motion as well as is involved in the transgenerational inheritance of learning ability, we expect to see the correlations between the strengths of neuronal activities and the efficiencies of learning ability in F1 mice cross-mated from HLE♂+HLE♀, HLE♀+LLE♂ and HLE♂+LLE♀ mice. To examine this possibility, we take the following parameters into our analysis. Learning efficiencies in the F1 mice of showing HLE, MLE and LLE are plotted in X-axis, including whisking frequency (Figure 7) and whisking angles (Figure 8) at training day six. The strengths of synaptic transmission (sEPSC and sIPSC amplitudes and frequencies) at 67% cumulative probability as well as the spikes induced by normalized stimuli at 3.0 in input-output curves on glutamatergic and GABAergic neurons in these F1 mice of showing HLE, MLE and LLE are plotted in Y-axis. As showed in Figures 7–8, learning efficiencies are linearly correlated to synaptic efficacy and spiking ability in these barrel cortical neurons from three groups of F1 mice. Thus, the cellular mechanism underlying the transgenerational inheritance of learning ability is based on the strengths of the upregulation at glutamatergic neurons and of the downregulation at GABAergic neurons.

## DISCUSSION

**Figure 9:**
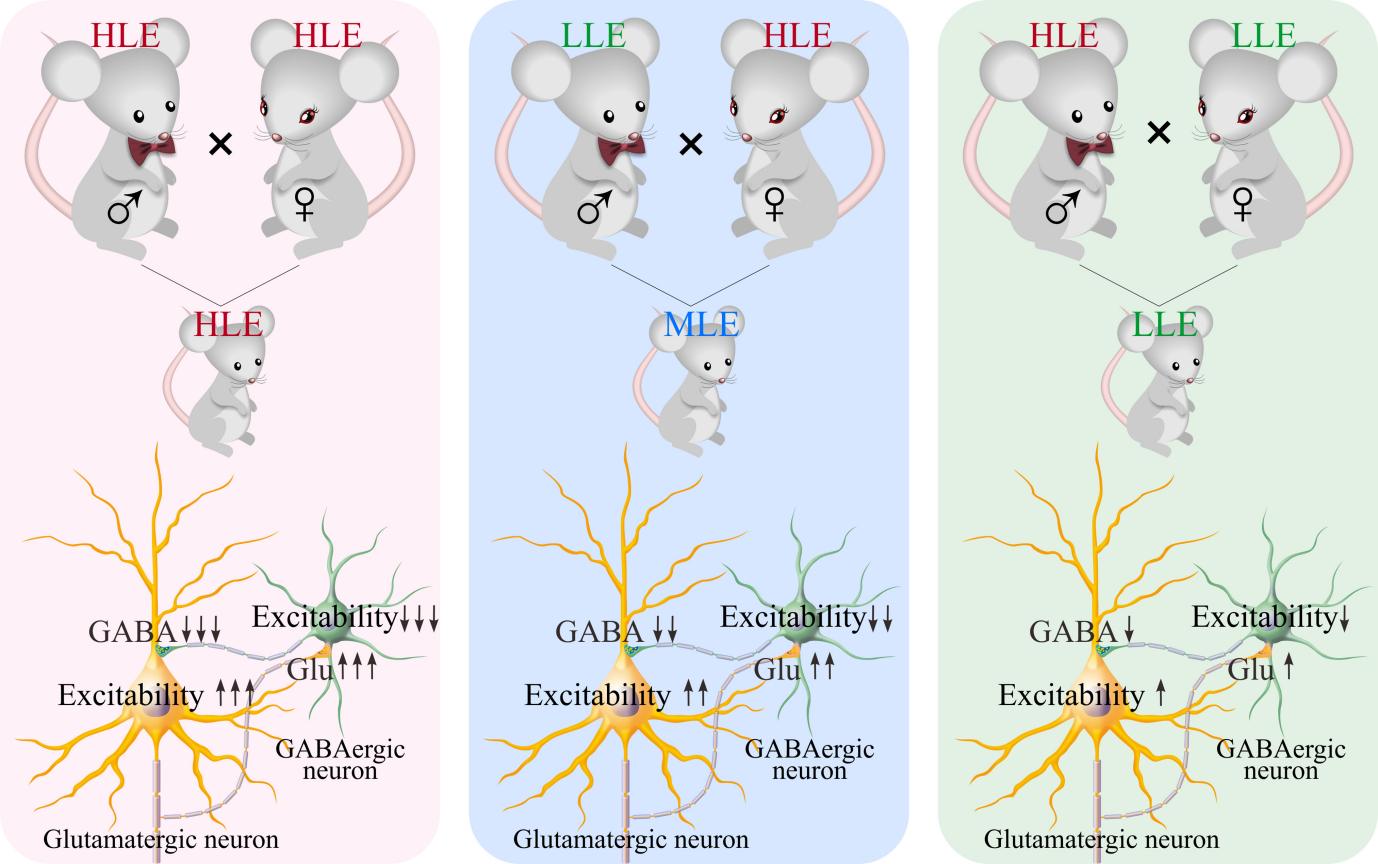
The strengths of plasticity at barrel cortical glutamatergic and GABAergic neurons are correlated with the efficiency of odorant-induced whisker motion in F1 mice Left panel shows neuronal plasticity coordinated between glutamatergic and GABAergic neurons in F1 mice cross-mated from HLE male and female parents. Middle panel shows neuronal plasticity coordinated between glutamatergic and GABAergic neurons in F1 mice cross-mated from HLE female and LLE male parents. Right panel shows neuronal plasticity coordinated between glutamatergic and GABAergic neurons in F1 mice cross-mated from HLE male and LLE female parents.

In mice that show odorant-induced whisker motion, i.e., a cross-modal associative memory, the ability of associative learning memory in parents can be transmitted to their filials. The parents with high learning efficiency reproduce the filials with high learning efficiency, in which the female appears in dominance, i.e., like mother like kids (Figure 1). In terms of cellular bases for this transgenerational inheritance of learning ability from F1 mice, barrel cortical glutamatergic neurons are upregulated in their excitatory synaptic transmission and spiking ability as well as are downregulated in their inhibitory synaptic transmission, especially those mice with high learning efficiency (Figures 2–4), while GABAergic neurons are downregulated in their excitatory synaptic transmission and spike ability (Figures 5–6). Furthermore, activity strengths in upregulated glutamatergic neurons and downregulated GABAergic neurons are linearly correlated with associative learning efficiency (Figures 7–8). Therefore, the upregulation of glutamatergic neurons and the downregulation of GABAergic neurons constitute the cellular basis for the transgenerational inheritance of learning ability (Figure 9), in which the upregulated glutamatergic neurons and the downregulated GABAergic neurons facilitate their recruitments to be associative memory cells [1, 30] and drive them to optimal state for information storages [17].

It is noteworthy that memory presentations i.e., the information retrievals shown by behaviors, are executed by neural circuits from sensory cortices to behavior-guide cortices through their relayed brain regions [1]. This suggestion is granted by the facts that stimulations to any of these areas can trigger memory retrievals [34–38] and that responses to associated signals can be recorded in sensory cortices [17, 22, 30] and their downstream brain areas [39–41]. Therefore, the sensory cortices are still primary locations for signal storage and retrieval initiation. This point is supported by seeing linear correlation between neuronal activity strength in sensory cortices and learning efficiency (Figures 7–8).

Learning and memory are thought to be hereditarily transmitted from parents to their filials. In addition to the transmissions of parent's cognitive behaviors directly to their filials, such as specific odor-induced fear memory [3] and anti-predation behavior [4], the ability of learning and memory may be transmitted from parents to their filials. Genetics and epigenetic regulation are thought to be molecular bases for the transgenerational inheritance of learning ability and memory to specific events [9–17]. The subcellular targets of genetic codes and epigenetic regulation for this transgenerational inheritance of learning ability remain unclear. Our results about the correlations between the efficiency of associative learning and the strength of coordinated neuronal activity in the filials provide one of cellular mechanisms for the transgenerational inheritance of learning ability. In other words, genetic- and epigenetic-regulated activity strengths between cortical glutamatergic and GABAergic neurons in the neural circuits influence the transgenerational inheritance of learning ability.

There is a linear correlation between learning efficiency and neuronal activity strengths in parents [42], which is consistent with this study that activity strengths of cortical glutamatergic and GABAergic neurons are correlated with transgenerational inheritance of learning ability in F1 mice (Figures 7–8). In other words, learning efficiencies in parents and their offsprings are correlated with neuronal activity strength. In addition to the similarity of cellular mechanisms, molecular mechanisms for these cellular regulations may be similar. In terms of molecular mechanisms underlying the correlation between the capability of associative memory and the strength of neuronal activities, both genetic and epigenetic processes are likely involved [9–17]. Genetic codes determine to build the structures of neural circuits and different type neurons as well as the functional states of these neurons mediated by ligand- and voltage--gated ion channels. In the transmission of learning and memory ability with high efficiency, these genetic codes guide the syntheses of molecular materials, which build neurons, synapses and glia cells, to organize their structures and set up the function of neural circuits and neurons well, such that the recruitments of associative memory cells and new synapses as well as the plasticity of these neural units are facilitated, or vice versa. In terms of the role of epigenetics in the transmission of learning ability, environment factors and learning processes may activate epigenetic processes to modulate the expression of the genetic codes and in turn to influence the building of neural units, the recruitments of associative memory cells and new synapses, as well as the plasticity of these units, such that the efficiency of learning and memory can be boosted well.

To a role of barrel cortical glutamatergic and GABAergic neurons in odorant-induced whisker motion with this transgenerational inheritance of associative learning ability, we propose that the upregulation of glutamatergic neurons and the downregulation of GABAergic neuros in the barrel cortex make these glutamatergic neurons to be more excitable, which permits the excitatory driving force from the new synapse innervations of the piriform cortex to recruit them as associative memory cells [17, 22, 30, 31] and to refine them with the upregulated ability to encode the digital spikes [43–45] for information storage. In terms of information retrievals, associative memory cells and their upregulations boost their ability to activate the neurons in the downstream brain areas for behavioral reactions and memory presentations. If the sensitivity and intrinsic property of these associative memory cells are increased, their driving to downstream neurons may lead to the elevated activity in brain areas related to behavior and emotion reactions for memory presentation, otherwise, memory extinction [1, 23]. If associative memory cells are over-excited, their pathological associations may be related to evoke illusion, delusion and convulsion [17].

In terms of the different regulations of glutamatergic and GABAergic neurons during associative learning, i.e., the reason why glutamatergic synaptic transmission and neuronal excitability are upregulated and GABAergic synaptic transmission and neuronal excitability are downregulated coordinately, our thoughts are given below. In the comparison with glutamatergic neurons, GABAergic neurons have low volume and high spiking ability, so that the high consumption as well as low energy storage and buffer volume of intracellular GABAergic neurons make them being vulnerable to high level activities [46, 47]. The active state of GABAergic neurons during associative learning makes them exhausted, such that their functions are downregulated. Moreover, intracellular signaling pathways may coordinate these changes since Ca^2+^ signaling coordinates the functions of different subcellular compartments [48].

There are two forms of transgenerational intelligence, the transmission of parent's cognitions and behaviors directly to their filials, such as specific odor-induced fear memory [3] and anti-predation behavior [4], as well as the transmission in the ability of learning and memory to their filials [5–8]. The transmission of specific memory from parents to their filials cannot rule out the maternal effects on filials, such as social learning [49, 50] after filials are born. The transmission of learning ability from parents to their filials also cannot rule out the postnatal training from parents to their filials, although the communications between parents and filials to tell the memorized experience are largely unknown. Whether the genetic codes, epigenetic processes or maternal effects are dominantly weight on the transgenerational transmission of learning and memory remains to be studied. Regardless of these influences, the strengths of neuronal activity may constitute the central point for this transgenerational feature of learning ability.

## MATERIALS AND METHODS

All experiments followed the guidelines by Administration Office of Laboratory Animals at Beijing China. All protocols were approved by Institutional Animal Care Unit Committee in Administration Office of Laboratory Animals at Beijing China (B10831).

### Mouse model of associative memory

C57 Thy1-YFP/GAD67-GFP mice were used [33], whose glutamatergic neurons were genetically labeled by yellow fluorescent protein and GABAergic neurons were labeled by green fluorescent protein. Experimental mice in F0 and F1 were selected based on their spontaneous activity and body weight in the consistent level [51].

Mice in postnatal days 20 were trained by the simultaneous pairing of mechanical whisker stimulus (WS) with odor stimulus (OS, butyl acetate toward the noses) by multiple-sensory modal stimulator (ZL201410499466) [17, 22, 30, 42]. The intensities, time and intervals of the OS and WS were precisely set (please see references [17, 22, 30]) . Each mouse in the home-made cage was trained twenty seconds in each time, five times per day with two hours of intervals for consecutively ten days. Cares were taken including no stressful condition and circadian to the mice that showed normal whisking and symmetric whiskers [17, 22, 30]. Long whiskers (such as arcs 1–2) on the same side and rows were assigned for mechanical stimuli and for the observation of their responses to the odor-test. This assignment was based on the studies in cross-modal sensory plasticity [52, 53]. We did not trim short whiskers because whisker trimming elevated barrel cortical neuronal excitability [33].

The traces of whisker motion were recorded by a digital video camera (240 Hz) and were quantified based on whisker retraction angle and whisking frequency (MB-Ruler, version 5.0 by Markus Bader, Germany). Whisking frequency was whisker fluctuation times per second. Whisking angles were measured as angles lined from original position to whisker retraction. The response of mouse whiskers to the odor-test (butyl acetate, 20 sec) was measured at the end of each training day to quantify the onset time and levels of odorant-induced whisker motion, conditioned response (CR). CR-formation needed to meet the following criteria. The patterns of odorant-induced whisker motion were similar to those of WS-induced whisker motion. Whisking frequency and angles significantly raised, compared to those before the training. As this type of whisker motion induced by odorant was originally induced by WS, the odor signal initiated a recall of whisker signal and led to whisker motion [17, 22, 30].

### Transgenerational analyses of odorant-induced whisker motion

Learning efficiency was measured based on the time at the full establishment of odorant-induced whisker motion. According to our data from all of the mice in the expression of odorant-induced whisker motion, we defined learning ability as either high or low efficiency. If their odorant-induced whisker motion reached to the plateau level before or at training day six, they were defined as the mice with high learning efficiency (HLE). If odorant-induced whisker motion reached to the plateau level at training day 10 or after, they were named as the mice with low learning efficiency (LLE). After the establishment of odorant-induced whisker motion, the associations of whisker and odor signals were given for one day per week to prevent the decay of this associative memory up to their cross-mating. In our studies, HLE male and female mice, HLE female mice and LLE male mice as well as HLE male mice and LLE female mice were cross-mated in inbred about three months postnatally, such that their filials in the first generation (F1) were classified into three groups, respectively. These F1 mice were trained by pairing whisker and odor stimulations to test their learning efficiency versus training days. Based on these dynamic curves, brain slices from these F1 mice were made at training day six, and their barrel cortical glutamatergic and GABAergic neurons were recorded to analyze the efficacy of synaptic transmission and the ability to convert excitatory inputs into digital spikes.

### Brain slices and neurons

The cortical slices (400 μm) were made from CR-formation F1 mice that were anesthetized by inhaling isoflurane and decapitated by a guillotine. The slices were sectioned by a Vibratome in the oxygenated (95%O_2_/5%CO_2_) artificial cerebrospinal fluid (ACSF), in which the chemical concentrations (mM) were 124 NaCl, 3 KCl, 1.2 NaH_2_PO_4_, 26 NaHCO_3_, 0.5 CaCl_2_, 4 MgSO_4_, 10 dextrose, and 5 HEPES, pH 7.35 at 4 °C. The slices were held in the oxygenated ACSF (124 NaCl, 3 KCl, 1.2 NaH_2_PO_4_, 26 NaHCO_3_, 2.4 CaCl_2_, 1.3 MgSO_4_, 10 dextrose, and 5 HEPES, pH 7.35) at 25°C for 2 hours, and then were transferred to a submersion chamber (Warner RC-26G) perfused with the oxygenated ACSF at 31°C for whole-cell recording [54].

Electrophysiological recordings on barrel cortical neurons in layers II-III were done under DIC-fluorescent microscope (Nikon FN-E600, Japan), in which the wavelength at 488 nm excited GFP and the wavelength at 575 nm excited YFP. GABAergic neurons appeared basket shape and fast spiking with less adaptation in spike amplitudes and frequency [55, 56]. Glutamatergic neurons showed pyramidal shape and regular spikes with adaptations of spike amplitude and frequency [51]. The cerebral slices were coronal sections including the barrels correspondent to the projection from long whiskers that were stimulated in paired-WS/OS training.

### Whole-cell recording

MultiClamp-700B amplifier in voltage-clamp or current-clamp was used to record neuronal electrical activities. Electrical signals were inputted into pClamp-10 (Axon Instrument Inc, CA USA) for data acquisition and analyses. Output bandwidth in this amplifier was 3 kHz. The pipette solution to study excitatory synapses included (mM) 150 K-gluconate, 5 NaCl, 5 HEPES, 0.4 EGTA, 4 Mg-ATP, 0.5 Tris-GTP and 5 phosphocreatine (pH 7.35; [57, 58]). The solution to study inhibitory synapses contained (mM) 130 K-gluconate, 20 KCl, 5 NaCl, 5 HEPES, 0.5 EGTA, 4 Mg-ATP, 0.5 Tris–GTP and 5 phosphocreatine [59]. Pipette solutions were freshly made and filtered (0.1 μm), osmolarity was 295–305 mOsmol and pipette resistance was 5–6 MΩ.

GABAergic neurons were functionally assessed based on active intrinsic properties and inhibitory outputs [47]. Inhibitory outputs were evaluated by recording spontaneous inhibitory postsynaptic currents (sIPSC) under a voltage-clamp on glutamatergic neurons in the presence of 10 μM 6-Cyano-7-nitroquinoxaline-2,3-(1H,4H)-dione (CNQX) and 40 μM D-amino-5-phosphonovanolenic acid (D-AP5) in ACSF to block ionotropic glutamate receptors [60, 61]. 10 μM bicuculline was washed onto slices at the end of experiments to test whether synaptic responses were mediated by GABA_A_R. sIPSCs were blocked by bicuculline in our experiments. Series and input resistances in all neurons were monitored by injecting hyperpolarization pulses (5 mV/50 ms), and calculated by voltage pulses versus instantaneous and steady-state currents. The pipette solution with the high level of chloride ions makes the reversal potential to be -42 mV. sIPSCs are inward when membrane potential is held at -65 [51, 61–63].

Glutamatergic neurons were functionally assessed based on active intrinsic property and excitatory outputs [47]. Excitatory outputs were evaluated by recording spontaneous excitatory postsynaptic currents (sEPSC) on GABAergic or glutamatergic neurons in presence of 10 μM bicuculline in ACSF to block ionotropic GABA receptors [47]. 10 μM CNQX and 40 μM DAP-5 were added into ACSF perfused onto the slices at the end of experiments to test whether synaptic responses were mediated by GluR. sEPSCs were blocked by CNQX and DAP-5 in our experiments. The series and input resistances for all cells were monitored by injecting hyperpolarization pulses (5 mV/50 ms), and calculated by voltage pulses versus instantaneous and steady-state currents [64].

Neuronal action potentials were induced by depolarization pulses, whose intensity and duration were altered based on the aim of experiments. The ability to convert excitatory inputs into digital spikes was evaluated by input-outputs (spikes versus normalized stimuli) when various stimulations were given [43, 48, 65–67], in which stimulus intensities were step-increasing by 10% normalized stimulations. As the excitability of different neurons was variable such that step-increased depolarization pulses were given based on their normalization. The base value of stimulus intensity for this normalization at each neuron was the threshold intensity of depolarization pulse (1000 ms in duration) to evoke a single spike [65].

Data were analyzed if resting membrane potential was negatively more than -60 mV and action potential amplitudes were more than 90 mV for GABAergic neurons, or if resting membrane potential was negatively more than -70 mV and action potential amplitudes were more than 100 mV for glutamatergic neurons. The criteria for the acceptance of each experiment also included less than 5% changes in resting membrane potential, spike magnitude, and input resistance throughout each experiment. In order to estimate the effects of associative learning on neuronal spikes and synaptic transmission, we measured sEPSC, sIPSC, input-output curves under the conditions of control and associative memory, which were presented as mean ± SE. The comparisons of sEPSCs and sIPSCs among the different groups were based on their values at 67% of cumulative probability.

### Statistical analyses

The paired *t*-test was used in the comparisons of the experimental data before and after associative learning as well as mouse whisking patterns in responses to whisker stimulus and odorant stimulus in each of the mice. One-way ANOVA was applied to make the statistical comparisons in the changes of neuronal and synaptic activities among CR-formation F1 mice with HLE, MLE and LLE; and two-way ANOVA with post hoc comparisons by *t*-test were used for Figures 1, 3 and 6.

## SUPPLEMENTARY MATERIALS FIGURES


