# Supplementary material for: Activity strengths of cortical glutamatergic and GABAergic neurons are correlated with transgenerational inheritance of learning ability
Source: Oncotarget. 2017 Aug 4;8(68):112401–16. doi: 10.18632/oncotarget.19918 (PMC5762519; doi:10.18632/oncotarget.19918)
Supplement: Supplementary file 1 [file oncotarget-08-112401-s001.pdf]

Activity strengths of cortical glutamatergic and GABAergic neurons are correlated with transgenerational inheritance of learning ability

SUPPLEMENTARY MATERIALS

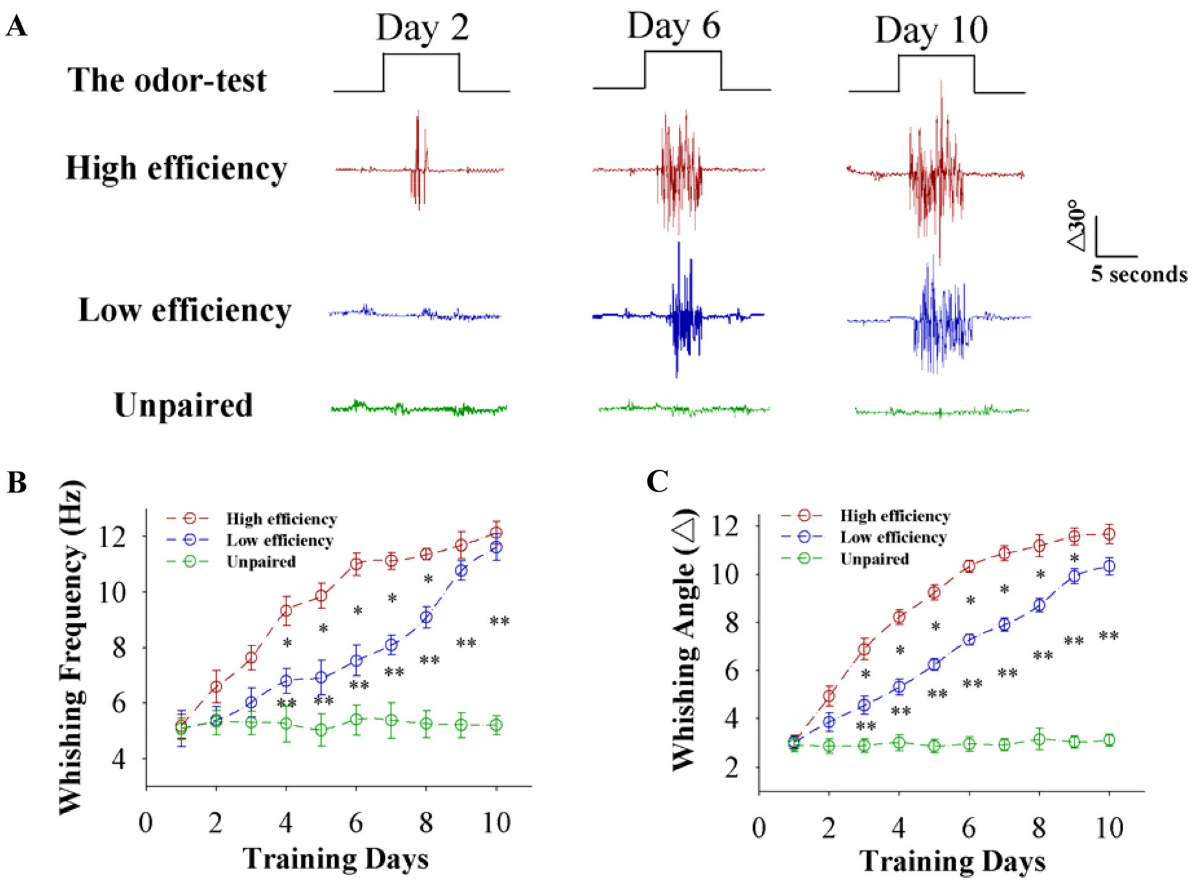

Supplementary Figure 1: Paired whisker and odor stimulations lead to odorant-induced whisker motion in the forms of high and low efficiency. (**A**) shows whisker motions in response to the odor-test pulses (black traces on top) in CR-formation mice with high efficiency (red traces) and low efficiency (blues) as well as an unpaired control mouse (greens) at training days two (left), six (middle) and ten (right). Calibration bars are 30o and 5 seconds. (**B**) shows whisking frequencies in response to the odor-test versus training days in CR-formation mice with high efficiency (red symbols) and low efficiency (blues) as well as an unpaired control mice (greens). (**C**) shows whisking angles in response to the odor-test versus training days in CR-formation mice with high efficiency (red symbols) and low efficiency (blues) as well as an unpaired control mice (greens). An asterisk shows $p < 0.05$ and two asterisks show $p < 0.01$, in which the statistical comparisons are two-way ANOVA with post hoc comparisons by *t*-test.

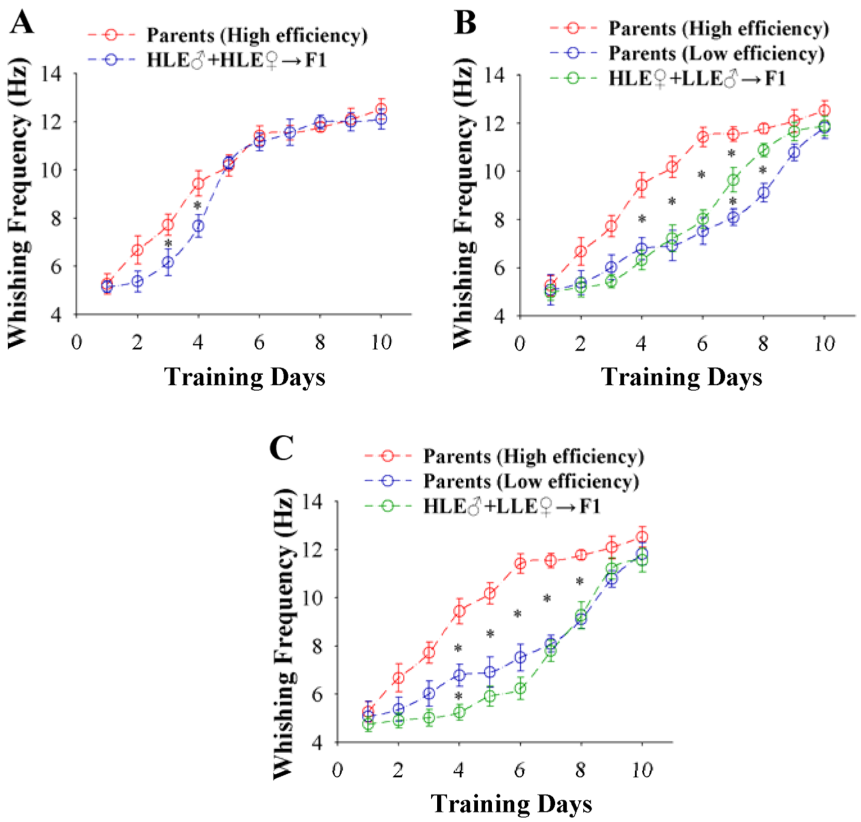

Supplementary Figure 2: Comparisons of odorant-induced whisker motion between Parents and F1 generations. (**A**) shows whisking frequencies in response to the odor-test versus training days of the parental high efficiency group (red) and the F1 mice from the cross-matings of HLE mice (HLE♂+HLE♀, blue). (**B**) illustrates whisking frequencies in response to the odor-test versus training days of the parental high efficiency group (red), the parental low efficiency group (blue) as well as the F1 mice from the cross-matings of HLE mice (HLE♀+LLE♂, green). (**C**) shows whisking frequencies in response to the odor-test versus training days of the parental high efficiency group (red), the parental low efficiency group (blue) as well as the F1 mice from the cross-matings of HLE mice (HLE♂+LLE♀, green). An asterisk shows $p < 0.05$, in which the statistical comparisons are two-way ANOVA with post hoc comparisons by *t*-test.
